# A single exercise bout enhances the manufacture of viral-specific T-cells from healthy donors: implications for allogeneic adoptive transfer immunotherapy

**DOI:** 10.1038/srep25852

**Published:** 2016-05-16

**Authors:** Guillaume Spielmann, Catherine M. Bollard, Hawley Kunz, Patrick J. Hanley, Richard J. Simpson

**Affiliations:** 1 Laboratory of Integrated Physiology, Department of Health and Human Performance, University of Houston, 3875 Holman Street, Houston, Texas 77204, USA; 2 School of Kinesiology, Louisiana State University, Baton Rouge, LA 70802, United States; 3 Program for Cell Enhancement and Technologies for Immunotherapy, Children’s National Health System and The George Washington University, Washington D.C., USA

## Abstract

Cytomegalovirus (CMV) and Epstein-Barr virus (EBV) infections remain a major cause of morbidity and mortality after allogeneic hematopoietic stem cell transplantation (HSCT). The adoptive transfer of donor-derived viral-specific cytotoxic T-cells (VSTs) is an effective treatment for controlling CMV and EBV infections after HSCT; however, new practical methods are required to augment the *ex vivo* manufacture of multi-VSTs from healthy donors. This study investigated the effects of a single exercise bout on the *ex vivo* manufacture of multi-VSTs. PBMCs isolated from healthy CMV/EBV seropositive participants before (PRE) and immediately after (POST) 30-minutes of cycling exercise were stimulated with CMV (pp65 and IE1) and EBV (LMP2A and BMLF1) peptides and expanded over 8 days. The number (fold difference from PRE) of T-cells specific for CMV pp65 (2.6), EBV LMP2A (2.5), and EBV BMLF1 (4.4) was greater among the VSTs expanded POST. VSTs expanded PRE and POST had similar phenotype characteristics and were equally capable of MHC-restricted killing of autologous target cells. We conclude that a single exercise bout enhances the manufacture of multi-VSTs from healthy donors without altering their phenotype or function and may serve as a simple and economical adjuvant to boost the production of multi-VSTs for allogeneic adoptive transfer immunotherapy.

Around 60,000 patients with genetic disorders and blood cancers receive allogeneic hematopoietic stem cell transplantation (HSCT) in the world each year^1^. While HSCT may be the best hope for their long-term disease free survival, the procedure is still associated with significant morbidity and mortality^2^. In particular, conditioning regimens required to deplete patient T-cells prior to engraftment delay immune reconstitution and leave the HSCT recipient vulnerable to potentially fatal viral infections. The ubiquitous herpesvirus cytomegalovirus (CMV) and Epstein-Barr virus (EBV) contribute substantially to these complications^3^, accounting for ~26% of all treatment-related deaths during the early post-transplant period^4^,^5^.

Adoptive transfer immunotherapy using donor-derived viral-antigen-specific cytotoxic T-cells (VSTs) has been shown to effectively prevent and control viral infections after HSCT^6^,^7^,^8^,^9^. VSTs are often directly isolated from donor blood samples using MHC class I multimers (i.e. pentamers or tetramers) that are loaded with synthetic virus specific peptide HLA molecules allowing them to bind to cognate receptors on the T-cells. However, this approach has limitations as it requires prior knowledge of immunodominant epitopes and is restricted by donor HLA type^10^. Furthermore, the HLA class I restriction in most commercially available multimers results in the selection of CD8+ but not CD4+ T-cells, which may limit the scope and duration of an immune response after transfer^10^. In contrast, selecting T-cells by their ability to secrete effector cytokines such as IFN-γ in response to viral peptide stimulation allows for the purification of many T-cell subtypes (from both CD8+ and CD4+ subsets) and is not restricted to certain HLA types or specific peptides. However, a limitation of both the multimer and cytokine capture methods is the low number of antigen-specific cells found in the circulation of healthy donors. This oftentimes results in insufficient numbers of antigen-specific T-cells being obtained from the donor to elicit adequate immune protection in the recipient after adoptive transfer.

The *ex vivo* expansion of VSTs have been found to be a viable alternative to cytokine capture and multimer-based selection methods^11^. Blood lymphocytes are typically taken from an HLA-matched healthy donor and expanded *in vitro* to recognize and kill cells infected with the target viral antigens. When sufficient numbers of VSTs are grown they are therapeutically transferred to the patient. Although the first method of generating VSTs was described over 20 years ago^12^, initially, prolonged manufacturing times were a problem taking 10–12 weeks to expand sufficient numbers of VSTs for adoptive transfer^6^,^13^. More recently, manufacturing times have been shortened to 1–10 days depending on the protocol^14^,^15^,^16^. However, using these rapid manufacturing protocols still requires a high frequency of circulating VSTs in peripheral blood to ensure the multi virus specificity of the final product. Moreover, inadequate restoration of antiviral immunity in some patients may be due to the failure to generate sufficient numbers of VSTs with broad virus specificity using these rapid manufacturing protocols^15^. Thus, new methods are required to increase the frequency of VSTs within the final product to be clinically efficacious.

The number of antigen-specific memory T-cells in the pre-expansion cell fractions is likely to underpin both the magnitude and the kinetics of the VST products generated *ex vivo*^17^. Although pre-sorting VSTs using HLA-peptides tetramers^12^ or antigen-responsive cells^18^ from blood prior to expansion may increase starting memory T-cell numbers and purity, this method is expensive, labor-intensive, and requires large and impractical T-cell numbers^19^,^20^. Gerdemann *et al*. showed that multi-VSTs could be manufactured in as little as two weeks by stimulating peripheral blood mononuclear cells (PBMC) with a combination of viral-antigen derived peptides and growth cytokines, and that the resulting VST products were both safe and effective^21^. Combining this later approach with a greater number of viral-specific T-cells in the starting PBMC fractions could dramatically improve the generation of polyclonal viral-specific T-cells for adoptive transfer immunotherapy.

One way to effectively and economically increase the number of starting antigen-specific T-cells in peripheral blood is through a single bout of dynamic exercise. It has been shown that individuals with a latent CMV infection mobilize twice as many CD8+ T-cells and ~4 times as many effector-memory CD8+ T-cells after exercise compared to their non-infected counterparts^22^,^23^, with many of the mobilized cells being specific to the CMV antigens IE-1 and pp65^22^. Moreover, the mobilized cells have broad CMV antigen epitope specificity^22^ and, in conjunction with our previous observations that exercise-mobilized cells are polyfunctional^24^ and have longer telomeres^25^, would indicate that the mobilization of VSTs with exercise may augment the *ex vivo* manufacture and functional properties of multi-VSTs for adoptive immunotherapy.

In this study, we exercised healthy donors previously exposed to CMV and EBV - viruses known to contribute to post-transplant morbidity and mortality. We found that a single bout of exercise dramatically augments the number of CMV and EBV-specific T-cells manufactured over 8 days, and that the resulting VSTs were capable of killing antigen-specific autologous target cells in an HLA-dependent manner. We conclude that exercise may serve as a simple and economical adjuvant to boost the number of multi-VSTs manufactured from healthy donors for use in the allogeneic adoptive transfer immunotherapy setting.

## Materials and Methods

### Participants

Serum samples obtained from potential participants were screened for both CMV and EBV antibody titers using commercially available ELISA kits (Biocheck inc., CA, USA and GenWay Biotech, CA, USA respectively), with 9 (3 female) otherwise healthy CMV and EBV IgG seropositive subjects being enrolled in the study. An additional male subject who was seronegative to both CMV and EBV was included as a control. All participants had above-average fitness levels in accordance with the American College of Sports Medicine (ACSM) age-adjusted scores for estimated maximal oxygen uptake (V∙O_2_max)^26^. They were non-smokers, not taking medication or supplements known to affect the immune system, and were free from any infectious illness (including conspicuous symptoms of herpesvirus reactivation) for 6-weeks prior to testing. Subjects were asked to fast overnight and refrain from exercise 24 hours prior to each laboratory visit. After receiving oral and written information pertaining to the risks and demands of the study, each subject provided their written informed consent. The study procedures were reviewed and approved by the Committee for the Protection of Human Subjects (CPHS) at the University of Houston in accordance with US federal regulations and the ethical principles established by the Belmont Report. The physical characteristics and exercise performance measures of the participants are presented in Table 1.

### Blood Lactate Threshold Testing and Experimental Exercise Protocol

All participants reported to the Laboratory of Integrated Physiology at the University of Houston between 07:30 and 09:00 hours and underwent an incremental discontinuous exercise test on a cycle ergometer (Velotron, Racermate, WA, USA) as previously described^27^. The curvilinear rise in lactate concentration on the graphical relationship between workload and blood lactate concentration was identified as the breakpoint blood lactate threshold (BLT) in accordance with the definition of Weltman^28^. The experimental exercise protocol was completed within 1 week after the BLT test and was conducted at the same time of day. For the experimental exercise trial, all subjects completed a 30-min steady state cycling protocol at a power output corresponding to 15% above their individual BLT. Heart rate was monitored continuously during exercise and rating of perceived exertion (RPE) was recorded every 5 minutes. Earlobe capillary blood samples were recorded every 10 minutes for blood lactate analysis. The physiological responses to the experimental exercise trial are shown in Table 1. Intravenous blood samples were collected from an antecubital vein into 6 ml vacuum tubes (containing either a serum gel separator or spray-coated with lithium heparin or EDTA) at rest (following 5 minutes of seated rest) and immediately after cessation of exercise. All blood samples were processed immediately to determine complete blood counts (Mindray BC3200, Mindray, NJ, USA) and isolate peripheral blood mononuclear cells (PBMCs) for phenotyping and VST generation. An additional resting blood sample was collected for phytohaemagglutinin (PHA) blasts generation for use in the autologous VST cytotoxicity assay.

### Peptide Stimulation and VST expansion

A total of 10 × 10^6^ freshly isolated PBMCs obtained before and after exercise were pelleted in a 15-mL centrifuge tube and pulsed for 60 minutes at 37 °C as previously described^21^. We used commercially available pools of CMV and EBV peptides (15mers overlapping by 11aa) (Pepmix™, JPT Peptide Technologies, Berlin, Germany). Each aliquot of 10 × 10^6^ PBMCs were pulsed with 400 ng of CMV-specific pp65, CMV-specific IE1, EBV-specific LMP2A and EBV-specific BMLF1 overlapping peptide libraries. Following incubation the pulsed cells were resuspended in 10 mL of RPMI 1640 supplemented with L-glutamine, 10% FBS and a combination of 10 ng/mL of IL-7 and 1,666 IU/mL of IL-4 (ebioscience, San Diego, CA, USA). The cells were then transferred to a gas-permeable cell culture flask (G-Rex10, Wilson-Wolf Manufacturing, New Brighton, MN) for 8 days at 37 °C. Media and cytokines were replenished at day 5 with fresh RPMI 1640 supplemented with 10% FBS, 10 ng/mL of IL-7, 1,666 UI/mL of IL-4 and 5 ng/mL of IL-15.

### T-cell phenotyping and subsets characterization

Freshly isolated PBMCs and the expanded VSTs (1.0 × 10^6^) were labeled with pre-diluted monoclonal antibodies (mAbs) in a 4-color direct immunofluorescence assay. Cells were incubated at room temperature in the dark for 45-minutes. The mAb combinations consisted of CD45RA FITC, CD62L PE, CD4 PerCP or CD8 PerCP and CD3 APC (ebioscience, San Diego, CA, USA). This allowed for the determination of naïve (NA; CD45RA+/CD62L+), central memory (CM; CD45RA−/CD62L+), effector memory (EM; CD45RA−/CD62L−) and CD45RA+ EM (EMRA; CD45RA+/CD62L−) subsets within the CD4+ and CD8+ T-cells^29^,^30^ before and after expansion. The phenotypes of the freshly isolated PBMCs (Day 0) and the expanded VSTs (Day 8) were assessed on a BD Accuri C6 flow cytometer (Accuri, Ann Arbor, MI, USA) as previously described^22^. The total number of T-cell subsets in blood before and after exercise was determined by multiplying the percentage of cells staining positive for the appropriate surface markers in the flow cytometry lymphocyte gate by the total blood lymphocyte count (Table 2).

### Intracellular cytokine staining

Freshly isolated PBMCs (Day 0) and the expanded VSTs (Day 8) were resuspended at 1.0 × 10^6^ cells per mL^−1^ and plated in 96 well plates (200 μL per well). The cells were then stimulated with 50 ng of PHA (positive control), RPMI alone (negative control) or viral peptides for 1 hour at 37 °C and for a subsequent 4 hours at 37 °C in presence of Brefeldin A (1 μg/mL). Following incubation, PBMCs and the expanded VSTs were washed twice with PBS and surface stained for 45 minutes with CD45RA FITC, CD62L PE and CD8 PerCP (ebioscience, San Diego, CA, USA) mAbs. The cells were then washed once more in PBS and fixed overnight at 4 °C in 4% Paraformaldehyde (Sigma Aldrich, St Louis, MO, USA). Excess fixating agent was washed off twice with permeabilization buffer (PBS+0.2% saponin) and cells were incubated for 45 minutes in permeabilization buffer and an anti-TNF-α mAb conjugated to APC (ebioscience, San Diego, CA, USA). Intracellular expression of TNF-α by the different T-cell subsets with and without peptide stimulation was analyzed on a BD accuri C6 flow cytometer.

### Flow cytometric Cytotoxicity assay

PHA blasts were generated for each subject by stimulating resting PBMCs with PHA (8 μg/mL) and IL-2 (400 UI) for 48–72 hours at 37 °C. After mitogen-induced expansion, the PHA blasts were cultured for 6 days, cryopreserved, and then thawed. After thawing, PHA blasts were incubated for 48 hours with IL-2 (200 IU) at 37 °C and then pulsed with CMV and EBV viral peptides. The cytotoxicity of the expanded VSTs was assessed by flow cytometry using a modified version of a previously published assay^31^. The PHA blasts were labeled with monoclonal APC-conjugated anti-CD3 for 45 minutes at 37 °C before being washed 3 times using RPMI 1640. The Expanded VSTs (effector cells) were incubated with the labeled PHA blasts for 4 hours at 37 °C at different concentrations (E:T ratios of 20:1, 15:1, 10:1 and 5:1) and target cells viability was determined by quantifying Annexin-V FITC and propidium iodide (eBioscience, San Diego, CA, USA) fluorescence on the CD3+ cells. Unpulsed PHA blasts along with PHA blasts pulsed with irrelevant peptides were used as a control for antigen specific cytotoxicity. Furthermore, some target cells were incubated with a pan unconjugated anti- MHC mAb for 45 minutes at 37 °C prior to CD3 labeling in order to assess the MHC-restricted killing capabilities of the VSTs.

### Enumeration of VSTs

Enzyme-linked immunospot (ELISPOT) analysis was used to enumerate CMVpp65, CMV IE1, EBV LMP2A and EBV BMLF1 in freshly isolated PBMCs and the expanded VSTs. PBMCs and VSTs were stimulated with the appropriate PepMixes at 37 °C for 12 h in an IFN-γ ELISPOT assay, with 100 000 PBMCs and 50 000 VSTs being stimulated with pepmixes at Day 0 and Day 8, respectively. Unstimulated PBMCs/VSTs and PBMCs/VSTs stimulated with PHA (1 μg/mL; Sigma-Aldrich) served as controls. Spot-forming cells (SFC) were enumerated by Zellnet Consulting Inc. (Fort Lee, NJ, USA) and calculated relative to input PBMC numbers before being adjusted for the number of CD3+ cells (% of PBMCs staining positive for CD3) in the PBMC fraction (IFN-γ SFC/input CD3+ T-cell number × 10^5^). This allowed us to express the data as the frequency of VSTs (SFC) per 100 000 T-cells. The total number of VSTs was also calculated by adjusting for the total number of CD3+ T-cells in the culture at Day 8 (SFC per T-cell × Total T-cell number). This allowed us to express the data as the total number of VSTs manufactured over 8 days of cell culture. Finally, the total number of VST subsets in whole blood before and after exercise was determined by multiplying the number of SFC/T-cell by the total blood T-cell count (Table 2).

### Statistical analysis

All data were assessed for assumptions of normality using the Shapiro-Wilk test and constant error variance prior to formal statistical testing. Skewed data were normalized by logarithmic transformation when required. Paired sample t-test were initially used to identify the exercise-induced mobilization of the different cellular subset at Day 0. Repeated measures ANOVA with two within factors (measurement day: 2 levels, and exercise-time: 2 levels) were used to compare the main effect of days in culture on the numbers of viral-specific T-cells and the interaction effect of days in culture with exercise-time (cells isolated from before and after exercise). The assumption of sphericity was tested using Mauchley’s method and post-hoc pairwise analyses were performed using Bonferroni adjustment. Paired T-tests comparing before (PRE) and after (POST) exercise within each measurement day (Days 0, 4 and 8) were used to identify differences in the phenotype of T-cells producing intracellular TNF-∝ after stimulation with viral peptides. All values are presented as the mean ± standard error of the mean (SEM). All statistical analyses were performed using “Statistical Package for the Social Sciences” (SPSS v17.0, Chicago, IL, USA) with statistical significance set at p < 0.05.

## Results

### Exercise evokes a lymphocytosis and preferentially mobilizes CD8+ T-cells with an effector memory phenotype

All participants successfully completed the 30-minute exercise bout. The effects of exercise on total leukocytes, granulocytes, monocytes, lymphocytes and T-cell subsets are presented in Table 2. The exercise bout elicited the archetypical leukocytosis with all major leukocyte subset numbers increasing POST. Total CD4+ and CD8+ T-cell numbers increased and all but the EMRA CD4+ and the CM CD8+ subsets were found to be elevated POST. As shown previously^22^, the single exercise bout evoked a greater mobilization (relative change in number from PRE to POST) of EM (t(9) = −4.1, p,0.05) and EMRA (t(9) = −3.5, p < 0.05) CD8+ T-cells compared to the NA and CM subsets (p < 0.05). Although exercise tended to increase the number of VSTs in the circulation, this did not reach statistical significance (p > 0.05). The relative mobilization of CD8+ T-cells was greater than CD4+ T-cells, but the reduction in the CD4:CD8 ratio POST did not reach statistical significance (p > 0.05).

### A single bout of exercise enhances the relative expansion of CD4+ and CD8+ T-cells responding to viral peptides without altering their subset composition

**Figure 1:**
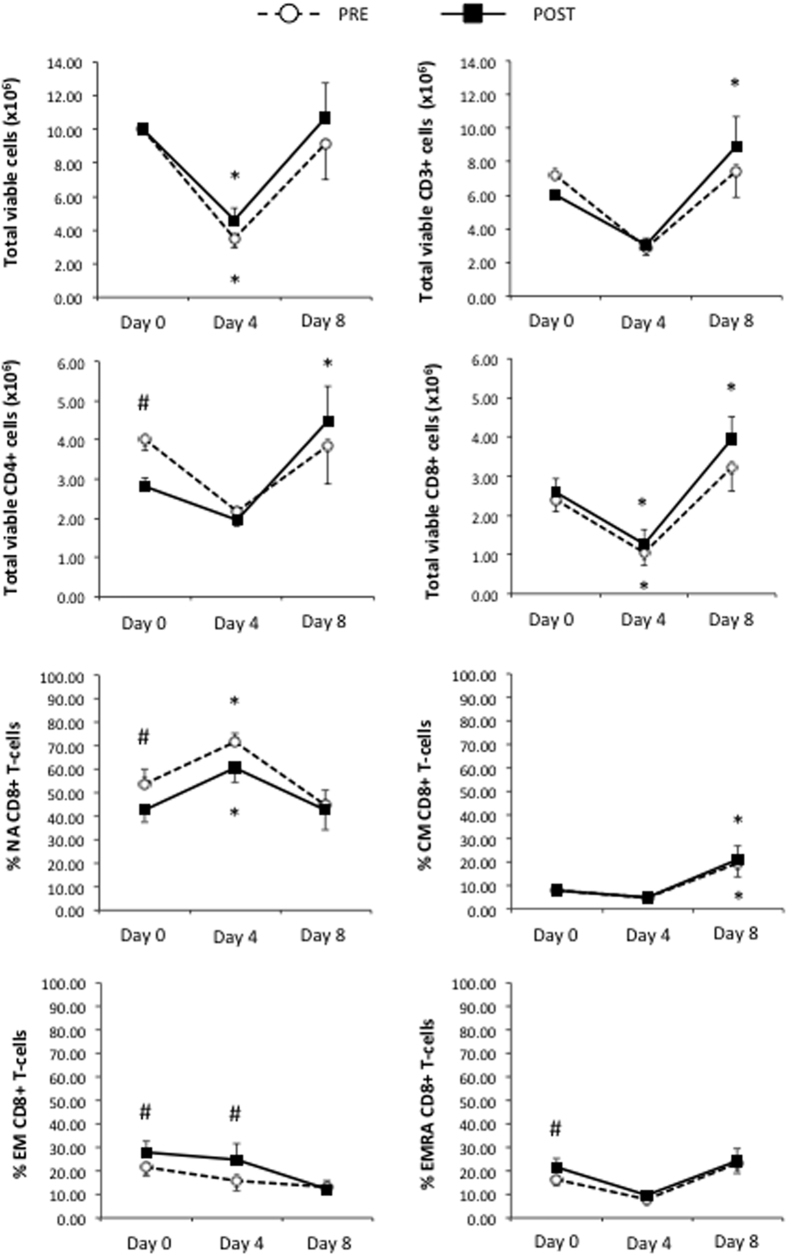
The effects of a single exercise bout on total T-cell numbers and CD8+ T-cell subset composition in response to viral-peptide stimulation over 8-days. PBMCs (10 × 10^6^) isolated before or after exercise were simultaneously stimulated with CMV pp65, CMV IE-1, EBV LMP-2 and EBV BMLF-1 peptides in the presence of IL-4 and IL-7. IL-15 was subsequently added at Day 4. Statistically significant difference from Day 0 indicated by (*p < 0.05). Statistically significant difference between the before (PRE) and after (POST) exercise conditions indicated by (#p < 0.05). Naïve (NA; CD45RA+/CD62L+), central memory (CM; CD45RA−/CD62L+), effector-memory (EM; CD45RA−/CD62L−) and RA+ effector-memory (EMRA; CD45RA+/CD62L−) T-cells.

A total of 10 × 10^6^ PBMCs collected PRE and POST were stimulated with a combination of 4 different overlapping peptide pools spanning entire regions of CMV (pp65 and IE1) and EBV (LMP2A and BMLF1) viral proteins. Following 8 days of culture in bioreactors, expanded viable T-cells were enumerated after anti-CD3, Annexin-V and Propidium Iodide staining. The total numbers of viable CD3+, CD4+ and CD8+ T-cells in resting and exercised cultures at Day 0, Day 4 and Day 8 are presented in Fig. 1. The total number of viable CD3+ T-cells was greater at Day 8 compared to Day 0 in POST (F(2, 7) = 8.022 p = 0.037) but not PRE (p > 0.05). This was due to a lower number of starting CD3+ cells at Day 0 in POST as there were no differences between PRE and POST at Day 8 (F(2, 7) = 3.278, p = 0.130). Moreover, exercise also increased the expansion of both CD4+ and CD8+ T-cells at Day 8 compared to Day 0 in POST but not PRE, but, again, the total cell numbers obtained at Day 8 did not differ between PRE and POST. As expected, exercise altered the composition of lymphocyte subsets at Day 0; reducing the number of CD4+ T-cells, increasing the proportion of EM and EMRA subsets among the CD8+ T-cells, and reducing the proportion of NA CD8+ T-cells. All proportional differences in CD8+ and CD4+ (data not shown) between the PRE and POST culture conditions at Day 0 were completely absent by Day 8.

Total cell numbers and CD8+ T-cell numbers decreased at Day 4 prior to the addition of IL-15 (IL-15 is not added during the first 4-days in order to deplete ‘contaminating’ NK-cells before expanding the VSTs) but the cell numbers did not differ between PRE and POST. Although proportional differences between PRE and POST remained at Day 4 for EM CD8+ T-cells, these were no longer present at Day 8. The proportions of CM CD8+ T-cells increased significantly during the last 4 days of culture (F(2, 7) = 5.461, p = 0.045) but by the same extent in both PRE and POST.

### Exercise enhances the *ex vivo* expansion of VSTs in response to viral peptide stimulation

**Figure 2:**
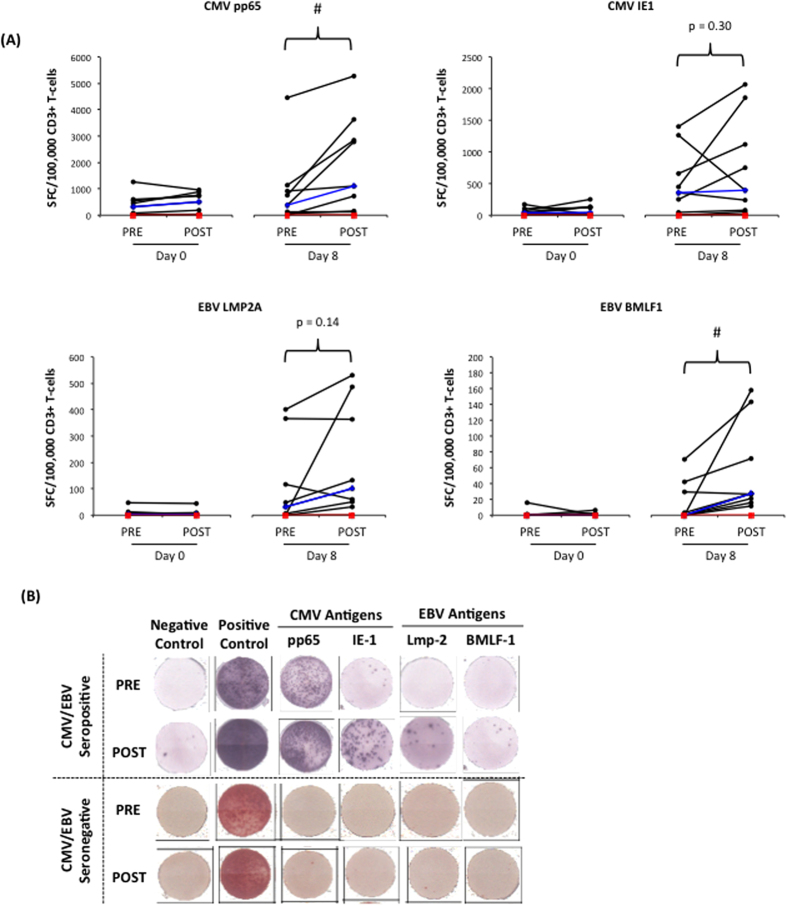
The effects of a single exercise bout on the frequency of viral-specific T-cells before (PRE) and after (POST) 8 days of viral-peptide stimulation. The numbers of CMV pp65, CMV IE-1, EBV LMP-2 or EBV BMLF-1 specific T-cells were enumerated before and after expansion in a 12 h IFN-γ ELISPOT assay. Spot forming cells (SFC) were adjusted per 100,000 CD3+ T-cell (as opposed to total cells) to account for the greater proportion of NK-cells among the PBMC fractions after exercise. Panel **A** shows individual subject responses for all CMV/EBV seropositive participants (n = 9). The median values are indicated by the blue line and the red line is the results from a CMV/EBV seronegative participant (n = 1). Statistically significant difference between the before (PRE) and after (POST) exercise conditions indicated by (^#^p < 0.05). Representative ELISPOT results for a CMV/EBV seropositive and seronegative participant are shown in Panel **B**.

Individual changes in the number of VSTs per 100,000 T-cells with exercise before and after expansion are shown in Fig. 2. We were not able to expand VSTs from a seronegative donor either before or after exercise. In the CMV/EBV seropositive donors, exercise did not affect the frequency of VSTs per 100,000 T-cells at Day 0 for any of the target antigens (p > 0.05). However, after 8 days of expansion, a greater number of CMV pp65-specific T-cells (F(1, 8) =  6.865, p < 0.05) and EBV BMLF1-specific T-cells (F(1, 8) =  6.532, p,0.05) were generated in the POST compared to the PRE condition. Additionally, a non-significant increase in the expansion of LMP2A-specific T-cells was observed in the POST condition compared to PRE (F(1, 8) = 2.678, p = 0.140). Exercise did not significantly affect the expansion of IE-1-specific T-cells (F(1, 8) = 1.2, p = 0.300).

**Figure 3:**
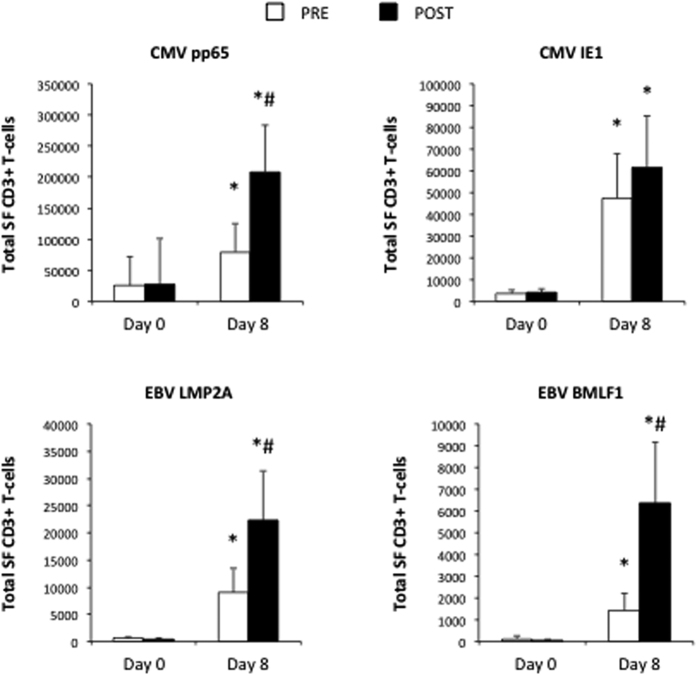
The effects of a single exercise bout on the frequency of viral-specific T-cells before and after 8 days of viral-peptide stimulation adjusted for total CD3+ T-cell numbers. To account for differences in total CD3+ T-cell numbers among PBMCs and the expanded VSTs, the proportion of viral-specific cells among PBMCs (Day 0) and the expanded VST’s (Day 8) were multiplied by the total number of CD3+ T-cells present in PBMCs and the expanded VST’s, respectively. Values are mean ± SEM. Statistically significant difference from Day 0 indicated by (*p < 0.05). Statistically significant difference between the before (PRE) and after (POST) exercise conditions indicated by (^#^p < 0.05).

As exercise tended to increase total T-cell expansions, we also expressed the VST expansion data relative to the total number of T-cells (VST/T-cell × total T-cells) recovered from the cultures at Day 8 (Fig. 3). The effects of exercise on the total number of VSTs generated was similar to the number of VSTs generated per 100,000 T-cells; however, the total number of EBV LMP2A-specific T-cells expanded POST was now significantly greater than the PRE condition [fold-difference: 2.5; F(1, 8) = 5.349, p < 0.05]. The effects of exercise on the expansion of CMVpp65 [fold-difference: 2.6; F(1, 8) = 6.073, p < 0.05] and EBV BMLF1 [fold-difference: 4.4; F(1, 8) = 5.652, p < 0.05) remained significant after adjusting for the total number of expanded T-cells. Although there was a trend for a greater expansion of total CMV IE1 VSTs in POST compared to PRE, this did not reach statistical significance (F(1, 8) = 1.448, p = 0.268).

### The phenotypic profiles of the expanded VSTs are not affected by exercise

**Figure 4:**
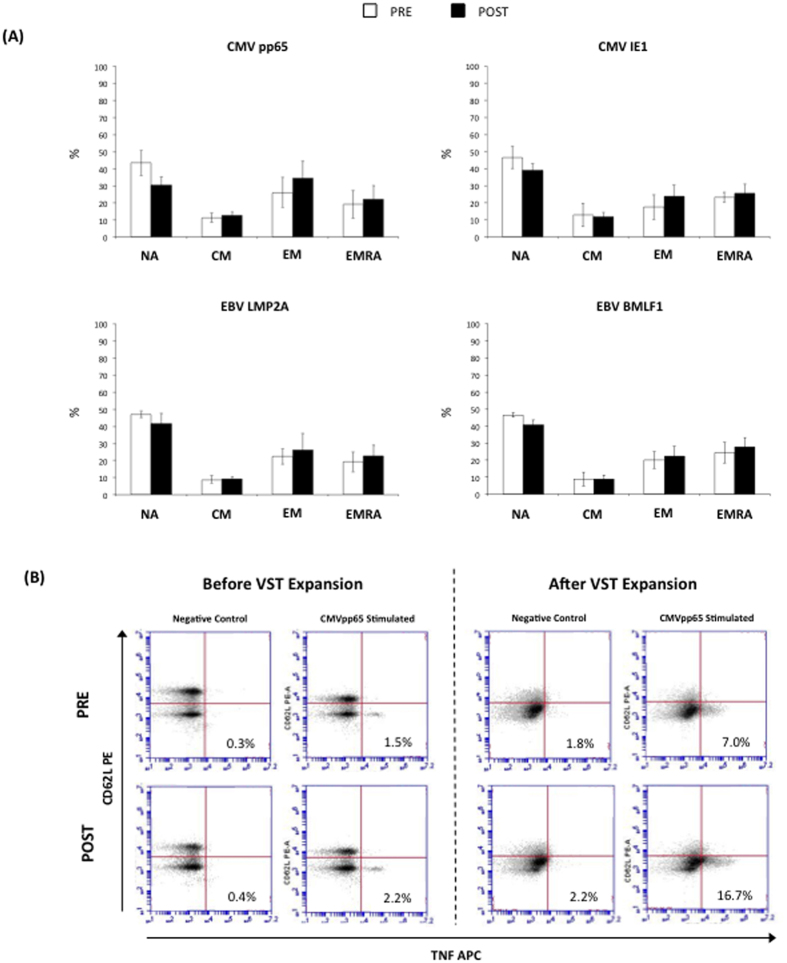
Panel A shows proportions (%) of naïve (NA), central memory (CM), effector memory (EM) and RA+ EM (EMRA) cells among CD8+ T-cells expressing TNF-α in response to viral peptide stimulation at Day 8. No significant differences in the composition of CD8+ T-cell subsets were found between the cells expanded before (PRE) or after (POST) exercise (p > 0.05). Panel **B** shows representative flow cytometry plots from the TNF-α intracellular cytokine staining assays following CMVpp65 stimulation or control (no peptides) of PBMCs obtained before (PRE) or after (POST) exercise prior to and after *ex vivo* expansion. Values in the lower right quadrants are expressed as the percentage of all CD8+ T-cells. The phenotypic characteristics of the peptide-responsive VSTs cells (Panel **A**) were determined through the co-expression of CD62L and CD45RA on the CD8+/TNF-α+ events by flow cytometry.

The VSTs were stimulated with individual peptides and the phenotypes of TNF-∝ producing cells were assessed by intracellular cytokine staining and flow cytometry (Fig. 4). No significant differences in the proportion of NA, CM, EM or EMRA VST-specific CD8+ cells were found between PRE and POST at Day 8 for any of the target antigens (p > 0.05).

### Cytotoxic activity of expanded VSTs is not affected by exercise

**Figure 5:**
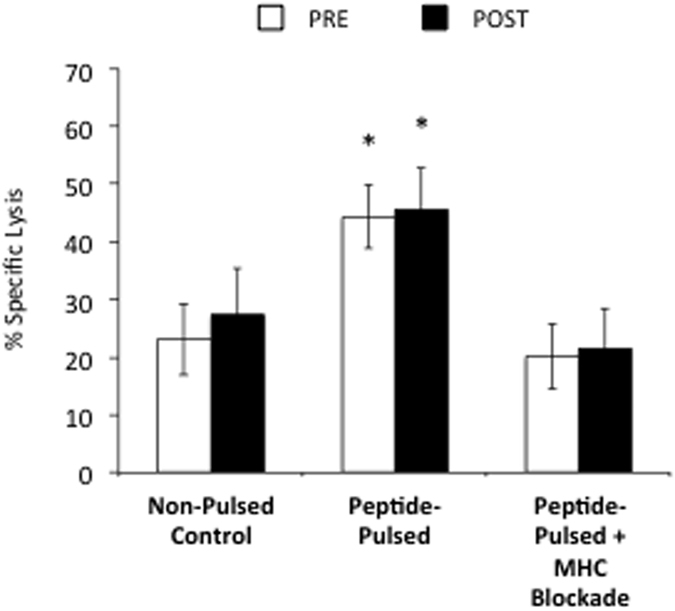
Antigen specific cytotoxicity of the CTL lines expanded before (PRE) or after (POST) exercise (n = 4). CTL lines expanded before or after exercise were co-cultured with autologous PHA blasts for 4 h under four separate conditions: (1) PHA blasts alone (control); (2) PHA blasts pulsed with the irrelevant viral peptides Hexon and Penton (n = 2; data not shown); (3) PHA blasts pulsed with all viral-peptides (CMV pp65, CMV IE-1, EBV LMP-2, and EBV BMLF-1); and (4) PHA blasts pulsed with all viral peptides in the presence of an anti-MHC monoclonal antibody (MHC blockade). *Indicates statistically significant difference compared to non-pulsed control and the peptide-pulsed condition with MHC blockade (p < 0.05). The non-pulsed control condition was not significantly different from the irrelevant peptide pulsed condition (data not shown; p > 0.05). No statistical differences were found between the CTL lines expanded before or after exercise for any condition (p > 0.05).

The cytotoxicity of the expanded VSTs was assessed using flow cytometry. Expanded cells (Day 8) were co-cultured for 4 hours with either autologous CD3+ cells (PHA blasts), autologous PHA blasts pulsed with the target viral-antigen peptides, or autologous PHA blasts pulsed with the target viral-antigen peptides following MHC blockade. The specific lysis of the PHA blasts by the expanded VSTs at an E:T ratio of 1:10 are presented in Fig. 5. Expanded VSTs in both PRE and POST killed the peptide-pulsed PHA blasts to a greater extent than the control condition (unpulsed PHA blasts). This effect was inhibited with MHC blockade, as the specific lysis of the petide-pulsed PHA blasts was no longer different from the unpulsed condition after labeling with a pan anti-MHC mAb. Thus, the VSTs expanded PRE and POST did not differ in their ability to kill peptide-pulsed autologous target cells in an MHC-restricted manner (t(3) = −0.753, p = 0.530).

## Discussion

There is a need to devise new practical methods to augment the frequency of virus-specific T cells for the manufacture of multi-VSTs from healthy donors for use in the allogeneic adoptive transfer immunotherapy setting. This study shows for the first time that a single bout of dynamic exercise markedly increases the production of CMV- and EBV-specific T-cells from healthy donors following a single exposure to clinically relevant viral peptides. The functional and phenotypic properties of the VSTs expanded after exercise did not differ from those expanded at rest, and were equally capable of recognizing and killing autologous target cells pulsed with viral peptides in an MHC-dependent manner. Exercise may therefore serve as a simple and economical adjuvant to boost the rapid generation of multi-VSTs for adoptive immunotherapy after HSCT.

We have shown previously that healthy adults carrying a latent CMV infection redeployed twice as many CD8+ T-cells than their non-infected counterparts, and that many of the mobilized cells were specific to the CMV antigens IE1 and pp65 and had broad epitope specificity^22^. We therefore hypothesized that the exercise-induced mobilization of these VSTs into the blood compartment would augment the production of cells lines with T-cells specific to multiple viruses for use in the allogeneic adoptive transfer immunotherapy setting. In the present study, we focused on CMV and EBV, two viruses known to cause significant morbidity and mortality after transplant, and elected to use the most rapid method available for generating multi virus-specific cytotoxic T-cells from healthy seropositive donors^21^. Using this expansion protocol on PBMCs collected immediately after 30 minutes of cycling, we were able to manufacture, on average, ~2.4x more CMV pp65, 3.1x more EBV LMP2A and 8.1x more EBV BMLF1 VSTs compared to stimulating the same number of PBMCs from resting blood. Although exercise did not significantly increase the production of CMV IE1 specific cells among the expanded VSTS compared to rest, this is likely due to the small sample size as 7 out of 9 subjects demonstrated a greater increase in IE1 specific VSTs after exercise (range: 0.7 to 4.2 fold different than the resting condition). Furthermore, while IE1 is an important target antigen during acute CMV infection^32^, the immune response to CMV is known to be mostly driven by the viral protein pp65^33^, leading to a greater number of pp65-specific T-cells compared to IE1-specific T-cells. Consequently, pp65 immunodominance may explain the lack of an exercise-effect on the expansion of IE-1-specific T-cells. The augmenting effects of exercise on VST manufacture appear to impact the antigen-specific memory T-cell compartment, as VSTs could not be expanded from a CMV and EBV seronegative participant either before or after exercise.

Although our hypothesis that exercise would augment the *ex vivo* manufacture of VSTs was supported, the effect does not appear to be due to numerical changes in the number of VSTs stimulated at Day 0. While exercise tended to increase the frequency of VSTs in the circulation per unit volume of blood, the proportion of VSTs among circulating CD3+ T-cells did not change with exercise. Consequently, the number of VSTs/CD3+ T-cell in PBMC fractions stimulated at day 0 did not differ between the pre and post exercise conditions. Despite this, both the total number of VSTs at Day 8 and the number of VSTs at Day 8 relative to Day 0 (Day 8 VST/Day 0 VST) was markedly greater after exercise compared to the resting condition, indicating that the exercise effect is not a result of numerical increases in circulatory VSTs. In fact, the total number of VST’s stimulated at Day 0 was actually less in the post exercise condition due to the substantially elevated proportion of NK-cells in the PBMC fractions. It seems unlikely therefore that the exercise effect reported here would be replicated under resting conditions by simply drawing larger volumes of blood. Moreover, not only is it impractical to obtain large volumes of blood from donors, it is also more expensive and laborious to run multiple cell cultures at once to accommodate the greater number of PBMCs from resting blood that would be required to generate VST numbers comparable with exercise.

The mechanisms by which exercise augments the *ex vivo* expansion of VSTs needs to be determined. Although exercise did not significantly increase the frequency of VSTs among circulating T-cells prior to expansion, it is possible that exercise preferentially mobilized ‘primed’ viral-specific T-cells, or enhanced the proliferative effects of *in vitro* cytokine supplementation. Indeed, exercise has been shown to increase plasma sIL2R concentrations^34^ along with STAT5 phosphorylation and subsequent IL-2R^35^,^36^, IL-7R^37^. Furthermore, acute bouts of exercise downregulate suppressor of cytokine signaling 3 (SOCS3) at the protein level^38^, leading to STAT3 activation, and overall increase in proliferative capacity. Consequently, although we initially hypothesized that cell shifts might drive the exercise-induced augmentation of VST expansion, it is possible that the 30-minute cycling bout led to germinal VSTs priming, ultimately increasing VST activation to both viral peptides and cytokine stimulation. It is also possible that the release of catecholamines, neurotransmitters, growth hormones and cytokines, with exercise could prime VSTs to increase their antigen responsiveness. Indeed, engagement of β_2_ adrenergic receptors is known to profoundly modulate the T-cell response by enhancing pro-inflammatory cytokine production when the stimulation precedes the antigenic challenge^39^. Furthermore, we also reported previously that exercise evokes the secretion of several cytokines, including IL-2, IFN-γ, TNF-α, IL-6, IL-4, and IL-10, even in the absence of exogenous stimuli and that this effect was localized to a differentiated subset of CD27−/CD8+ T-cells^24^. Altered cell subset proportions could also be responsible for the augmenting effects of exercise on *ex vivo* VST generation. For instance, exercise is known to lower the proportion of regulatory T-cells among PBMCs^40^ that could otherwise suppress T-cell proliferation *in vitro*^41^.

The present findings indicate that exercise can dramatically augment the manufacture of VSTs using clinically applicable peptides, which is advantageous in itself because they allow for the production of a single cell line with simultaneous specificity for multiple antigens, while also eliminating the reliance on complex manufacturing processes that require infectious virus material, production of a clinical grade vector, and overly long cell culture processing (10–12 weeks)^6^,^13^. It has been predicted that multi-VSTs expanded after viral peptide exposure will be safe to infuse and will provide broad-spectrum antiviral protection without increasing the risk of GvHD^21^. Combining exercise with this approach will not only increase the number of VSTs among the expanded cell lines, but might also reduce the time taken to generate sufficient numbers of VSTs for infusion. We also showed that the expanded virus peptide-specific cells had similar phenotypic characteristics and subset composition regardless of whether they were manufactured before or after exercise, and that both VST lines were equally effective at killing autologous peptide-pulsed targets via the MHC. This indicates that exercise does not augment the function of the expanded VSTs at the individual cell level, but rather increases the number of fully functional multi-virus specific cells within the expanded VST product.

The benefits of exercise might go beyond *ex vivo* expansion protocols and also improve other methods for purifying and selecting VSTs from donor blood samples such as donor lymphocyte infusions, and multimer isolation and cytokine capture methods. This may allow for the direct transfer of sufficient numbers of allogeneic VSTs without the need for *ex vivo* expansion and the effects of exercise on these methods should be investigated in future studies. Although the optimal dose of VSTs necessary to effectively treat active infections post HSCT remains to fully elucidated, small clinical studies have shown that infusing 0.5 to 2 × 10^7^ VSTs per m^2^ was sufficient to resolve active infections in 80% of patients^14^. Whether or not these thresholds can be reached more efficiently with exercise using both direct isolation and *ex vivo* expansion methods is an area for future investigation.

The methods and exercise protocols used here should be well suited to clinical application. Although 30 minutes of cycling exercise at +15% of the individual blood lactate threshold is considered ‘vigorous’ exercise, the majority (6/10) of subjects were able to complete the cycling protocol within a heart rate range of 75–85% of their age-predicted maximum heart rate (220-age). The ACSM recommends that healthy adults engage in steady state exercise that elicits a heart rate response of 65–85% of the maximum heart rate for 20–60 minutes, 3–5 times/week in order to improve/maintain aerobic fitness^42^. This would indicate that the exercise protocol used in the present study is achievable by most healthy individuals with above average cardiorespiratory fitness levels. However, future studies should determine the minimum exercise-intensity and duration threshold required to augment the manufacture of VSTs so that the application can be broadened to the wider HSCT donor population, including those with below average fitness levels. It also remains to be determined if exercise can augment the *ex vivo* manufacture of VSTs from individuals with below average fitness levels.

A limitation of the present study is that we did not determine the cytokine profiles or epitope specificity of the VSTs expanded before and after exercise. Manufacturing VSTs that recognize multiple epitopes is important to minimize virus escape due to epitope loss and to produce sustained anti-viral immunity *in vivo* following adoptive transfer^21^. However, due to our previous observations that exercise redeploys polyfunctional T-cells that constitutively secrete many cytokines, particularly among the differentiated (CD27−) CD8+ subsets^24^, and that CMV-specific cells mobilized with exercise have broad epitope specificity^22^, we would expect the VSTs produced after exercise to be at least comparable to those generated from resting blood. It is also possible that exercise, due to its augmenting effects on VST expansion, could drive T-cells closer to their maximum proliferative capacity prior to infusion, which would limit further expansion of the cell product *in vivo* after transfer. We deem this unlikely, however, as we have shown previously that average telomere lengths among CD8+ T-cells mobilized by exercise are longer compared to those present in resting blood^25^ indicating that exercise-mobilized T-cells have a longer clonal lifespan. It will be important to show that exercise does not evoke T-cell exhaustion and futures studies should examine PD-1 expression on the cell products expanded before and after exercise. Another potential limitation is our use of non HLA-restricted peptides, which would have allowed us to determine if the augmenting effects of exercise are related more to CD4+ or CD8+ T-cells, or indeed other affected cell populations among PBMCs. It also remains to be seen if exercise can augment the manufacture of other VSTs without causing unmanageable ‘cytokine storms’ or antigen competition. Gerdemann *et al*.^21^ found no evidence of antigen competition using pooled viral peptides to produce a single VST line with reactivity for 15 antigens derived from 7 latent and lytic viruses (EBV, CMV, BK, HHV6, Adv, Flu and RSV); however, Papadopoulou *et al*.^15^ showed that *in vitro* VST expansion polarized the final product towards the most immunodominant peptides. It is possible that the more robust CMV pp65-specific T-cell response evoked by exercise could polarize the VST expansions and inhibit the specificity and avidity of those T-cell products designed to react with multiple antigens. Future work will determine if exercise can reliably augment the manufacture of single T-cell lines with broad specificity and avidity to multiple viral antigens beyond those derived from just CMV and EBV.

In summary, this is the first study to demonstrate that a single bout of exercise markedly augments the *ex vivo* manufacture of CMV- and EBV-specific T-cells from healthy donors following a single exposure to clinically relevant viral peptides and growth cytokines over 8-days of cell culture. The VSTs produced after exercise were equally effective at killing autologous peptide-pulsed target cells, and had similar phenotype characteristics to those T-cell lines produced from the same donors at rest. This builds on our previous findings that exercise can increase the *ex vivo* production of tumor-antigen reactive T-cells^43^ and monocyte-derived dendritic cells^44^, indicating that acute exercise has broad applicability in the manufacture of cell products that are useful for immunotherapy. Further studies are required to determine if the augmenting effects of exercise are due to preferable changes in the extracellular milieu, the mobilization of more responsive VSTs, or to proportional shifts in the composition of cells within the PBMC fraction exposed to peptides. We conclude that a single bout of dynamic exercise is a simple and economical adjuvant to boost the rapid *ex vivo* manufacture of multi-VSTs for allogeneic adoptive transfer immunotherapy.

## Additional Information

**How to cite this article**: Spielmann, G. *et al*. A single exercise bout enhances the manufacture of viral-specific T-cells from healthy donors: implications for allogeneic adoptive transfer immunotherapy. *Sci. Rep.* **6**, 25852; doi: 10.1038/srep25852 (2016).

## Figures and Tables

**Table 1:** Physical characteristics and exercise performance measures of the participants (n = 10; 2 females).

Characteristics	Mean	SD	Range
Age (yrs)	31.1	3.8	26–37
Height (cm)	172.3	7.9	158–179
Mass (kg)	73.2	13.5	52.2–92.0
BMI (kg m^−2^)	24.5	3.4	21.8–30.7
Cycling power at blood lactate threshold (watts)	130.5	32.0	75–170
Physical activity rating (0–7)^a^	6.1	1.0	5.0–7.0
Estimated V∙O_2_max (mL.kg^−1^.min^−1^)^b^	45.4	3.2	40.1–49.4
Exercise Measures^c^			
Cycling power (w)	150	37.4	85–195
Average heart rate (beats min^−1^)	158.6	5.5	151–170
Average heart rate (% of estimated maximum)^d^	85.9	2.9	81.0–91.4
Average Blood lactate (mM)	3.7	0.9	1.9–4.8
Average Rating of perceived exertion (Borg 6–20 Scale)	15.3	1.5	14–17

^a^Determined using the physical activity rating scale: infrequent (0–1), moderate (2–3) and vigorous (4–7) physical activity^45^.

^b^Estimated using the Jackson *et al*. non-exercise equations^45^.

^c^All measures collected continuously (i.e. heart rate) and periodically (i.e. blood lactate; RPE) were averaged over the 30-minute exercise bout.

^d^Maximum heart rate estimated by the equation: 191.5 − (0.007 × age^2^)^46^.

**Table 2:** Total and differential leukocyte counts, total T-cell subset (CD4+ and CD8+) counts, total CD4+ and CD8+ subset counts, the CD4:CD8 T-cell ratio, and the total number of T-cells responding to CMV (pp65; IE-1) and EBV (LMP-2; BMLF-1) antigens in peripheral blood before (PRE) and immediately after (POST) 30-minutes of steady state cycling exercise (n = 10).

Cell Subset (cells/μL)	PRE	POST
Leukocytes	5538 ± 1121	9888 ± 2205 #
Lymphocytes	2006 ± 237	4075 ± 808 #
Monocytes	425 ± 158	1025 ± 103 #
Granulocytes	3163 ± 903	4875 ± 153 #
CD3+ T-cells	1465 ± 171	2491 ± 569 #
CD4+ T-cells	840 ± 181	1201 ± 274 #
*CD4+ NA*	468 ± 157	613 ± 238 #
*CD4+ CM*	265 ± 63	391 ± 110 #
*CD4+ EM*	99 ± 66	162 ± 98 #
*CD4+ EMRA*	18 ± 31	43 ± 67
CD8+ T-cells	450 ± 126	968 ± 465 #
*CD8+ NA*	251 ± 66	401 ± 106 #
*CD8+ CM*	36 ± 14	79 ± 38
*CD8+ EM*	73 ± 51	211 ± 172 #
*CD8+ EMRA*	89 ± 61	277 ± 226 #
CD4:CD8 Ratio	2.01 ± 0.72	1.52 ± 0.73
CMV pp65 SFC	4.8 ± 5.8	10.2 ± 10.8
CMV IE1 SFC	0.7 ± 0.8	1.6 ± 1.9
EBV LMP2A SFC	0.1 ± 0.2	0.2 ± 0.3
EBV BMLF1 SFC	0.03 ± 0.08	0.03 ± 0.07

CD4+ and CD8+ T-cell subsets were identified as naïve (NA; CD45RA+/CD62L+), central memory (CM; CD45RA−/CD62L+), effector-memory (EM; CD45RA−/CD62L−) and RA+ effector-memory (EMRA; CD45RA+/CD62L−) cells. The number of spot forming cells (SFC) enumerated in the ELISPOT assay at Day 0 following CMV or EBV peptide stimulation was adjusted for the total blood CD3+ cell count. Statistically significant difference from PRE indicated by (^#^p < 0.05). Data are mean ± SD.
